# A prognostic index based on an eleven gene signature to predict systemic recurrences in colorectal cancer

**DOI:** 10.1038/s12276-019-0319-y

**Published:** 2019-10-02

**Authors:** Seon-Kyu Kim, Seon-Young Kim, Chan Wook Kim, Seon Ae Roh, Ye Jin Ha, Jong Lyul Lee, Haejeong Heo, Dong-Hyung Cho, Ju-Seog Lee, Yong Sung Kim, Jin Cheon Kim

**Affiliations:** 1 Personalized Genomic Medicine Research Center, Korea Research Institute of Bioscience and Biotechnology, Daejeon, 34141 Korea; 2 Department of Cancer Research, Institute of Innovative Cancer Research and Asan Institute for Life Sciences, Asan Medical Center, Seoul, 05505 Korea; 3 Genome Editing Research Center, Korea Research Institute of Bioscience and Biotechnology, Daejeon, 34141 Korea; 4 Department of Surgery, University of Ulsan College of Medicine, Seoul, Korea; 5 Graduate School of Life Sciences, Kyungpook National University, 41566 Daegu, Korea; 6 Department of Systems Biology, Division of Cancer Medicine, The University of Texas MD Anderson Cancer Center, Houston, Texas United States of America

**Keywords:** Cancer genomics, Prognostic markers, Colorectal cancer

## Abstract

Approximately half of colorectal cancer (CRC) patients experience disease recurrence and metastasis, and these individuals frequently fail to respond to treatment due to their clinical and biological diversity. Here, we aimed to identify a prognostic signature consisting of a small gene group for precisely predicting CRC heterogeneity. We performed transcriptomic profiling using RNA-seq data generated from the primary tissue samples of 130 CRC patients. A prognostic index (PI) based on recurrence-associated genes was developed and validated in two larger independent CRC patient cohorts (*n* = 795). The association between the PI and prognosis of CRC patients was evaluated using Kaplan–Meier plots, log-rank tests, a Cox regression analysis and a RT-PCR analysis. Transcriptomic profiling in 130 CRC patients identified two distinct subtypes associated with systemic recurrence. Pathway enrichment and RT-PCR analyses revealed an eleven gene signature incorporated into the PI system, which was a significant prognostic indicator of CRC. Multivariate and subset analyses showed that PI was an independent risk factor (HR = 1.812, 95% CI = 1.342–2.448, *P* < 0.001) with predictive value to identify low-risk stage II patients who responded the worst to adjuvant chemotherapy. Finally, a comparative analysis with previously reported Consensus Molecular Subgroup (CMS), high-risk patients classified by the PI revealed a distinct molecular property similar to CMS4, associated with a poor prognosis. This novel PI predictor based on an eleven gene signature likely represents a surrogate diagnostic tool for identifying high-risk CRC patients and for predicting the worst responding patients for adjuvant chemotherapy.

## Colorectal cancer: a tool to identify risk of recurrence

A prognostic tool that searches for eleven genes associated with colorectal cancer recurrence shows promise in initial trials. The complexity of colorectal cancer (CRC) makes it challenging to treat. A significant number of patients relapse or experience metastasis even after surgical intervention, and fail to respond to post-surgical chemotherapy. Yong Sung Kim at the Korea Research Institute of Bioscience and Biotechnology, Daejeon, and Jin Cheon Kim from the University of Ulsan, Seoul, South Korea, and co-workers, conducted RNA-sequencing analysis on samples from 130 patients with CRC, 58 of whom had suffered relapse. They pinpointed eleven genes strongly associated with recurrence-free survival. They used these to develop a prognostic tool to identify high-risk patients and those more likely to respond poorly to chemotherapy. The tool was validated on a further 795 patients with CRC.

## Introduction

Colorectal cancer (CRC) is the third most common cancer and fourth leading cause of cancer death worldwide^1^. Although pathological staging is the gold standard for the prognosis and determination of adjuvant chemotherapy treatment in CRC^2^, few studies are available as a predictive tool for stage-specific recurrence. A large number of CRC patients relapse after complete surgical resection, and approximately half of patients with stage III disease relapse within 5 years^3,4^. International guidelines for the adjuvant treatment of stage II CRC recommend a range of treatment options from observation to chemotherapy with single-agent or combination regimens, depending on the presence of high-risk features, such as poorly differentiated histology, lymphovascular invasion, perineural invasion, retrieval of <12 lymph nodes, bowel obstruction, localized perforation, or positive margins^5^. Several prospective investigations have been carried out to address the role of adjuvant chemotherapy in stage II CRC, but the value of adjuvant therapy remains controversial^5^. The current National Comprehensive Cancer Network guidelines do not clearly define high-risk stage II colon cancer, as many patients with high-risk features do not have recurrence, whereas some patients are deemed to be average-risk^6^.

However, according to the report from the World Health Organization (WHO: http://www.who.int/cancer/en/), colon cancer is one of the most prevalent types of cancer in both sexes, and 50% of patients with this type of cancer will develop metastases during the course of the disease, primarily in the liver, lungs, or both. Currently, metastatic resection of CRC in patients with isolated liver and/or lung metastasis remains the only option for potential cure^6,7^. However, even when resection is combined with modern adjuvant systemic regimens, this treatment is curative in only 20% of patients and mostly fails due to tumor progression and dissemination. Therefore, a better molecular understanding of CRC is urgently needed for early and accurate patient prognostication, enabling precision medicine for cure^8^.

Recurrence can be divided into two types, namely, locoregional and systemic. Locoregional recurrence is defined as disease occurring at the anastomosis, mesentery or nodal basin, or in the peritoneum^9^. Here, we investigated putative genetic indicators associated with systemic recurrence, which occupy most recurrences regardless of the curative potential of surgery, in CRC using multiple cohorts. To explore all possible genes associated with systemic recurrence in CRC, we applied a genome-wide survey of transcriptome data and attempted to distinguish subgroups of CRC with distinct biological characteristics associated with CRC recurrence. Through experimental assays, we also validated a strong association between the gene signature and CRC recurrence.

## Materials and methods

### Patients and tissue samples

We shared the RNA-seq data from our previous study of tissue samples obtained from only the primary site of the colon or rectum in 130 CRC patients (as a training set) to find differentially expressed genes and their functions in terms of the pathologic characteristics of lymphovascular and perineural invasion^10^. All patients were treated at the Asan Medical Center (AMC, Seoul, Korea) between 2008 and 2015, including 72 and 58 patients without and with systemic recurrences, respectively (Supplementary Table S1). In patients with stage II disease, postoperative adjuvant chemotherapy after curative surgery was administered by using 5-FU/leucovorin in the risk group, and sometimes by using FOLFOX in younger patients. Otherwise, the use of FOLFOX, FOLFIRI, and biologics alone or in a combined regimen for advanced stage III and IV patients is recommended. Postoperative adjuvant chemotherapy or postoperative chemoradiotherapy was administered as previously indicated^11^. In this study, systemic recurrence was considered as CRC recurrence with systemic disease and as metastatic CRC at diagnosis (i.e., Stage IV).

All tumor samples were acquired after individual patient consent for tissue sample donation and examination was received. The study protocol was approved by the Institutional Review Board of Asan Medical Center (registration no. 2018-0087), in accordance with the Declaration of Helsinki.

### RNA extraction, RNA-seq experiments, and data processing

RNA extraction and RNA-seq experiments were carried out as previously described^12^. Reference genome sequence data for *Homo sapiens* were obtained from the National Center for Biotechnology Information (NCBI) genome database (assembly ID: GRCh38). Kallisto was applied to the tissue samples for mapping and quantifying the reads to the reference genome (ver. 0.43.1). To estimate gene expression levels, transcripts per million (TPM) mapped reads values were calculated for each sample. The TPM data were normalized using quantile normalization in the R language environment (version 3.2.5). The sequencing coverages of RNA-seq data in the AMC cohort are described in Supplementary Table S2. The datasets generated by RNA-seq are available in the NCBI Gene Expression Omnibus public database under the data series accession numbers GSE50760 and GSE107422.

### Public datasets of CRC patients

To develop and validate a classifier for predicting disease recurrence in CRC, we collected two large publicly available datasets of CRC patients: gene expression data were collected from the Cartes d’Identité des Tumeurs (CIT) Programme from the French Ligue Nationale Contre le Cancer (CIT cohort, GSE39582, *n* = 566)^13^, and gene expression data were generated from fresh-frozen tumor specimens retrieved from the tissue banks of the Royal Melbourne Hospital, Western Hospital, Peter MacCallum Cancer Center in Australia and the H. Lee Moffitt Cancer Center in the USA (AUS cohort, GSE14333, *n* = 229)^14^. All gene expression data of the CIT and AUS cohorts were generated using the Affymetrix Human Genome U133 Plus 2.0 platform. The details of these patient cohorts have been described in previous literature^12^, and the baseline characteristics of all patient cohorts, including the AMC cohort, are illustrated in Supplementary Table S1. In this study, disease-free survival, defined as the time from surgery to the first confirmed relapse, was considered the primary endpoint. An adequate survival analysis could not be performed in our training cohorts according to the profound inclination of stage IV patients (44.6%).

### Statistical analysis

To identify a gene list highly associated with systemic recurrence in CRC, we applied a point-biserial correlation test to the transcriptome data from the AMC cohort and selected genes having significant correlation coefficients with disease recurrence (|*r*| > 0.3 and *P* < 0.001). A hierarchical clustering analysis, with the centered correlation coefficient as a measure of similarity and complete linkage clustering method, was performed using a gene expression data matrix consisting of recurrence-associated genes. Based on sample clusters, the CRC patients were dichotomized into two subgroups, between which differences in frequencies of disease recurrence were assessed using Fisher’s exact test. A functional enrichment analysis was performed using the Ingenuity Pathway Analysis tool.

To develop a prognostic index (PI) for each patient with CRC, we adopted a previously developed strategy using the Cox regression coefficient for the genes from the CIT cohort^12,15,16^. The PI of each patient was calculated as the sum of each gene score, which was derived by multiplying the expression level of a gene by its corresponding coefficient (i.e., PI = ∑ Cox coefficient of gene *G*_*i*_ × expression value of gene *G*_*i*_). The patients were then divided into two groups (i.e., high- or low-risk of recurrence) using the median cut-off of the risk score as a threshold. The coefficient of each gene obtained from the CIT cohort was directly applied to data from the AUS cohort to divide the patients into high- and low-risk groups.

The Kaplan–Meier method was used to calculate the time to recurrence, and the difference in survival between the two groups was assessed using log-rank statistics. The prognostic association between the PI and known clinicopathological factors was assessed using multivariate Cox proportional hazard models. A backward-forward step procedure was applied to optimize the multivariate model with the most informative variables^17^. To estimate the significance of gene expression differences between subgroups, two-sample *t*-tests were performed for each gene. The R language environment software (ver. 3.5.1) was mainly used for statistical analyses, in which the survival analysis was performed using the APPEX platform^18^.

For comparative analysis between subgroups displayed by the PI and the previously reported consensus molecular subtypes (CMS)^19^, we adopted a CMS classifier provided by the CMS consortium (https://www.synapse.org/#!Synapse:syn4961785) to stratify the CRC patients into four CMS subtypes in the AMC and AUS cohorts. In the case of the CIT cohort, CMS classification data were available as supplementary material in the investigation, which was applied in a comparative analysis in this study^19^.

### Real-time reverse transcriptase polymerase chain reaction (RT-PCR) analysis

To determine the association between the genes and systemic recurrence of CRC, RNA (1 μg) was isolated from CRC patients excluded in the AMC cohort using an RNeasy Plus Mini Kit (QIAGEN #74136) according to the manufacturer’s instructions. The isolated RNA samples were reverse-transcribed with Superscript II reverse transcriptase (Invitrogen, Carlsbad, CA) for 1 h at 42 °C and for 5 min at 85 °C and amplified using quantitative RT-PCR with the primer sets described in Supplementary Table S3. For quantitative RT-PCR, 100 ng of cDNA was analyzed using 2 × SYBR Green Supermix (Bio-Rad, Hercules, CA) and the CFX96TM optics module (Bio-Rad Inc., Foster City, CA) with the following amplification protocol: 95 °C for 3 min, followed by 40 cycles at 95 °C for 20 s, annealing at (56–60 °C) for 20 s and extension at 72 °C for 20 s. The gene encoding *β-actin* was amplified as a control. The relative quantification of target mRNA was analyzed by the comparative threshold cycle (Ct) method.

## Results

### Identification of two distinct prognostic subtypes in CRC by transcriptomic profiling

**Fig. 1:**
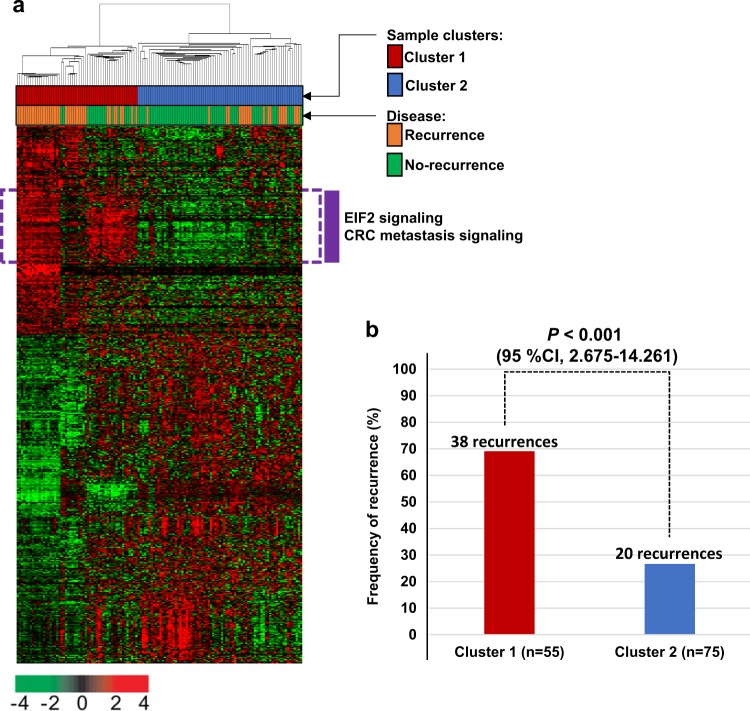
Gene expression profile of the systemic recurrence-associated genes in the AMC cohort (*n* = 130). **a** A total of 1160 genes with expression patterns that significantly correlated with disease recurrence in colorectal cancer (CRC) were selected for cluster analysis (point-biserial correlation test, *P* < 0.001, *r* < −0.3 or *r* > 0.3). By hierarchical clustering, the patients were divided into two subclusters. **b** Comparison of the frequencies of recurrence in cluster 1 and cluster 2. *P*-values and confidence intervals (CIs) were obtained using Fisher’s exact test

We first evaluated a correlation between systemic recurrence and gene expression using the data from the AMC cohort. When the correlation coefficients between disease recurrence and the gene expression levels were estimated (point-biserial correlation test, *P* < 0.001, |*r*| > 0.30), a total of 1160 gene features with a change in expression that correlated with systemic recurrence were identified. Using these genes, we performed a hierarchical clustering analysis to classify the CRC samples into two subclusters (cluster 1 and 2; Fig. 1a). When comparing the rates of systemic recurrence between the two-sample clusters, the recurrence rate in cluster 1 (69.09%, 38 of 55) was significantly higher than that in cluster 2 (26.67%, 20 of 75). The difference in disease recurrence between the CRC clusters was statistically significant (Fisher’s exact test, odds ratio 6.049, 95% confidence interval (CI) = 2.675–14.261, *P* < 0.001; Fig. 1b). Exploring the expression pattern of 1160 genes, we discovered a distinct subset of genes that was more highly expressed in sample cluster 1 than in cluster 2 (152 genes surrounding by a dashed line of purple color in Fig. 1). Among these genes, many genes involved in the EIF2 or CRC metastasis signaling pathways were significantly enriched (Fig. 1), indicating that our newly discovered CRC subtypes might reflect distinct molecular characteristics of poor-prognostic CRC.

### Molecular characteristics of the two prognostic CRC subtypes

To explore the biological characteristics that are active during CRC recurrence, a functional enrichment test of the 1160 recurrence-associated genes was performed. When searching for significantly associated previously established pathways, not surprisingly, the genes involved in the EIF2 and CRC metastasis signaling pathways were significantly enriched (Supplementary Fig. S1), consistent with the previous observation (Fig. 1). Along with these pathways, a number of important canonical pathways, such as the IL-6, AMPK, ERK/MAPK, IL-8, JAK/Stat, PDGF, mTOR, and CDK5 signaling pathways, were significantly activated in the poor-prognostic sample cluster 1. In contrast, the interferon, PTEN, apoptosis, p53, and ATM signaling pathways were significantly inhibited in cluster 1, signifying that various biological processes might closely affect the aggressive clinical behavior of CRC (Supplementary Fig. S1). When aggregating the genes included in these pathways, only 127 out of 1160 genes were involved (Supplementary Table S4), whereas the remaining genes were not associated. We applied these 127 genes in the subsequent downstream analyses.

### Development of a PI system and its validation

**Fig. 2:**
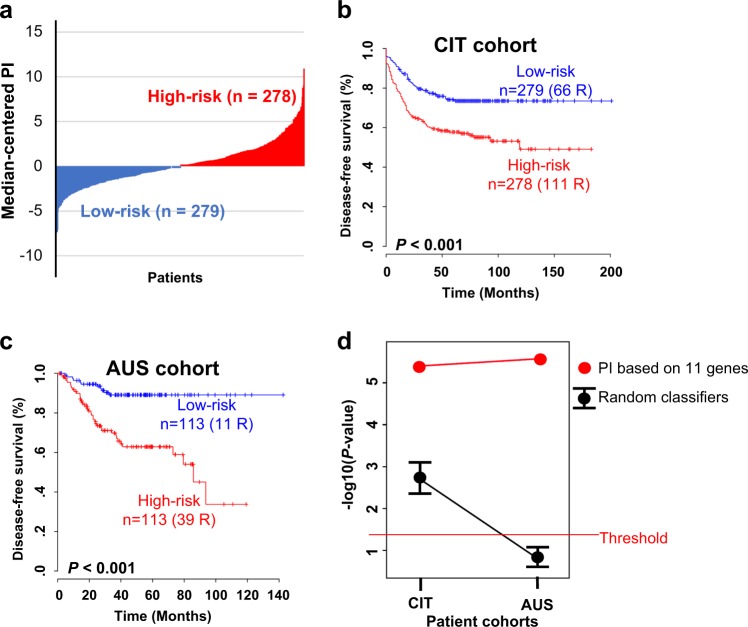
Colorectal cancer (CRC) patient stratification with the prognostic index (PI). **a** Bar-plot of the PI based on the 11 gene signature in the CIT cohort. Each bar indicates the risk score for an individual patient. **b** Kaplan–Meier curves showing the time to recurrence in the two subgroups in the CIT cohort stratified by the 11 gene-based PI. **c** Kaplan–Meier curves of the two subgroups in the AUS cohort stratified by the PI derived from the CIT cohort. **d** Comparison between randomly generated classifiers and the 11 gene-based PI. Confidence interval (CI) plots of *P*-values obtained using the log-rank test in random classifiers are displayed. Each dot indicates the average *P*-value. The upper and lower bars indicate the upper and lower values of the 95% CI for significance, respectively. The 95% CI was obtained from 1000 resampling events. Each red dot indicates the significance level of the PI classifier. All values were expressed on a -log10 transformed scale, and the threshold of significance was *P* < 0.05

To generate an optimal predictive model of CRC recurrence, we evaluated the prognostic value of the 127 genes in additional CRC cohorts. When applying a Cox proportional hazard model to the expression of each gene and the recurrence-free survival of CRC patients in the CIT cohort, only 11 genes from 17 unique probes showed strong prognostic relevance for CRC recurrence (Cox regression analysis, *P* < 0.01; Supplementary Table S5). The PI of each patient in the CIT cohort was calculated using the Cox regression coefficient of each of the 11 genes. Using the median cut-off of PI, the CRC patients in the CIT cohort were divided into the high-risk or low-risk subgroups (Fig. 2a). The recurrence rate in high-risk patients classified by the PI was significantly higher than that in the low-risk subgroup (log-rank test, *P* < 0.001; Fig. 2b). To validate the PI system, the coefficient values derived from the CIT cohort were directly applied to the gene expression data from the AUS cohort, in which the patients were dichotomized into high-risk and low-risk groups. The recurrence rates were significantly different between the two subgroups in a log-rank test analysis (*P* < 0.001; Fig. 2c).

To verify whether the PI system showed a significant prognostic value by chance, we generated 11 gene-based random classifiers and compared their prognostic significance with the PI. With the same procedure used for the development of the PI, we created a classifier with 11 randomly selected genes from 1160 genes (Fig. 1) and confirmed its prognostic significance. We iterated the generation of the random classifier 1000 times. Compared with the PI system and randomly generated classifiers, the significance levels of the PI in both the CIT and AUS cohorts were out of the CI range for significance in the case of random classifiers (Fig. 2d), indicating that the PI was not generated by chance.

To further verify an association between the PI and systemic recurrence in CRC, we also applied an RT-PCR analysis to the additional CRC samples, independently of the AMC cohort. Among the 11 genes incorporated into the PI, four (*EIF4A2*, *ITGB1*, *RHOC*, and *BID*), which showed a more statistically significant difference in expression between the recurrence and no recurrence subgroups in the AMC cohort, were evaluated in this analysis. When comparing the expression levels of the genes between 11 CRC patients with disease recurrence and 8 CRC patients with no recurrence, three genes (i.e., *EIF4A2*, *ITGB1*, and *RHOC*) showed significant differences in expression between the recurrence subgroups and revealed positive correlations with the RNA-seq data in the AMC cohort (Supplementary Fig. S2a-c). The difference in expression of *BID* between RT-PCR samples was not statistically significant, but the change in gene expression was positively correlated with the disease recurrence events in the AMC cohort (Supplementary Fig. S2d). Overall, these results supported an association between genes involved in the PI and systemic recurrence in CRC.

### Assessment of the relationship between known clinical factors and the eleven gene-based PI system for recurrence in CRC

**Fig. 3:**
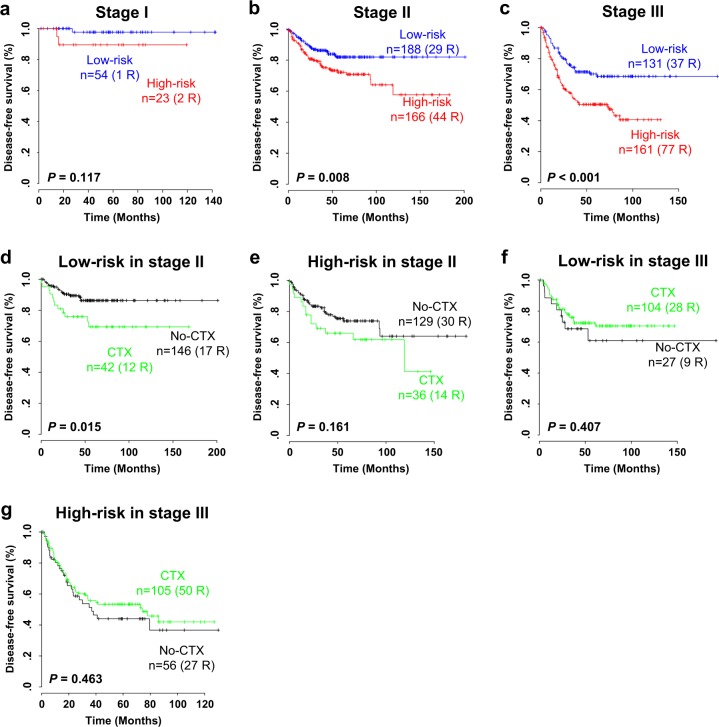
Kaplan–Meier plots of disease-free survival (DFS) of patients grouped by AJCC stage in the cohort combined with the CIT and AUS cohorts (*n* = 795). **a–c** Prognostic index (PI)-based subset analysis in (**a**) stage I, (**b**) stage II, and (**c**) stage III patients. The PI predictor was predictive in patients at stage III. *P*-values were obtained by log-rank tests. **d–g** Prediction of the response to chemotherapy in the two subgroups based on the PI predictor. Kaplan−Meier plots of stage II patients in the PI classifying (**d**) low-risk and (**e**) high-risk subgroups, and stage III patients in the PI classifying (**f**) low-risk and (**g**) high-risk subgroups are illustrated. The PI score showed a significant predictive value in (d) low-risk stage II CRC patients for adjuvant chemotherapy. The data were plotted according to whether patients received chemotherapy (CTX). *P*-values were obtained by log-rank tests

Staging is a gold standard for determining the prognosis of CRC patients^20^. To investigate the prognostic independence of the PI, we combined the clinical data from the CIT and AUS cohorts and performed a subset analysis according to the AJCC staging system. When stratification based on the PI was applied to stage I–III patients in the combined cohort, the population of high-risk patients with stage II and III diseases showed a significantly poorer prognosis in recurrence than that of the low-risk patients (*P* = 0.008 in stage II and *P* < 0.001 in stage III; Fig. 3b, c). Although a clear tendency toward classifying high-risk CRC patients in recurrence was observed at stage I, we did not observe any statistically significant difference in disease recurrence between subgroups (*P* = 0.117; Fig. 3a). To further estimate the independent prognostic utility of the PI, we also applied Cox regression analyses to the PI and known clinicopathological risk factors in CRC. In the univariate analysis, significant prognostic indicators of CRC recurrence included the AJCC stage, tumor location, chemotherapy, CMS subtype, and PI (Table 1). When the multivariate analysis was performed, the PI still illustrated statistical significance for disease recurrence, even after applying a variable selection procedure (hazard ratio (HR) = 1.812, 95% CI = 1.342–2.448, *P* < 0.001; Table 1). Among the validation cohorts, DNA mismatch repair (MMR) data were available only in the CIT cohort, in which we further applied Cox regression analyses to the PI and other risk factors, including the MMR status. In the univariate and multivariate analyses using the CIT cohort, the PI robustly showed statistical significance for disease recurrence (HR = 1.709, 95% CI = 1.194–2.447, *P* = 0.003; Supplementary Table S6), demonstrating the prognostic relevance of the PI system as an independent risk predictor for CRC recurrence.

**Table 1:** Univariate and multivariate Cox regression analysis of disease-free survival in colorectal cancer (combined with CIT and AUS cohorts)

Variable	Univariate			Multivariate			Multivariate (*step*^a^)		
	*n*	HR (95% CI)	*P*-value	*n*	HR (95% CI)	*P*-value	*n*	HR (95% CI)	*P*-value
Gender (male vs. female)	782	0.803 (0.617–1.047)	0.105	721	0.78 (0.583–1.045)	0.096	721	0.783 (0.587–1.045)	0.097
Age (<75 or ≥75)	782	0.986 (0.736–1.321)	0.927		1.203 (0.858–1.685)	0.283			
AJCC Stage (I, II, III, or IV)	782	2.837 (2.334–3.447)	<0.001		2.267 (1.781–2.885)	<0.001		2.327 (1.877–2.886)	<0.001
Location (distal, proximal, or rectum)	782	0.765 (0.602–0.973)	0.029		0.809 (0.622–1.053)	0.114		0.823 (0.636–1.066)	0.14
Chemotherapy (No or Yes)	766	1.841 (1.404–2.414)	<0.001		1.101 (0.789–1.536)	0.572			
CMS subtype (CMS1, 2, 3, or 4)	736	1.274 (1.123–1.446)	<0.001		1.134 (0.992–1.296)	0.066			
PI (low-risk or high-risk^b^)	782	1.984 (1.514–2.6)	<0.001		1.794 (1.327–2.426)	<0.001		1.812 (1.342–2.448)	<0.001

*HR* hazard ratio, *CI* confidence interval, *CMS* consensus molecular subtype, *PI* prognostic index

^a^A backward-forward step procedure was applied to optimize the multivariate model with the most informative variables

^b^Predicted outcome in Fig. 2b, c was used for analysis

Because adjuvant chemotherapy data were available in the combined cohort, we examined whether the PI could predict CRC patients who would benefit or not from adjuvant chemotherapy. The analysis was performed on AJCC stage II patients (*n* = 353), for whom adjuvant treatment is controversial^5^, and stage III patients (*n* = 292), who experienced prolonged survival following adjuvant chemotherapy^21,22^. When the stage II patients were divided into high- and low-risk subgroups based on the PI and the difference in disease recurrence was independently assessed, the low-risk stage II patients treated with adjuvant chemotherapy showed a significantly poorer clinical response than that of those without treatment (*P* = 0.015, Fig. 3d), while no association was observed in the high-risk stage II patient subgroup (*P* = 0.161, Fig. 3e). When the Cox regression model was applied to stage II patients, the interaction of the PI with adjuvant chemotherapy reached a statistically significant level (*P* < 0.001, Supplementary Fig. S3a). Consistent with the Kaplan–Meier plot and log-rank test, the estimated HR for adjuvant chemotherapy in the low-risk group was 2.426 (95% CI = 1.158–5.081; *P* = 0.018), retaining a significant predictive value, while the HR in the high-risk group was 1.571 (95% CI = 0.832–2.967; *P* = 0.163). In another subset analysis of stage III patients, adjuvant chemotherapy clearly improved disease-free survival in patients in the high- and low-risk patient subgroups, but it did not show any statistical significance (each *P* > 0.05, Fig. 3f, g). We also applied a Cox regression model to stage III patients to examine whether the PI was interactive with adjuvant chemotherapy. In this test, the interaction of the PI with adjuvant chemotherapy reached a statistically significant level (*P* = 0.034), but no significant predictive value of the PI classification in the adjuvant chemotherapy of stage III patients was discovered (Supplementary Fig. S3b). Taken together, the PI system showed significant prognostic potential in both stage II and III CRC patients, particularly to determine adjuvant chemotherapy in stage II CRC patients.

### Comparison of the CMS with the PI in CRC

**Fig. 4:**
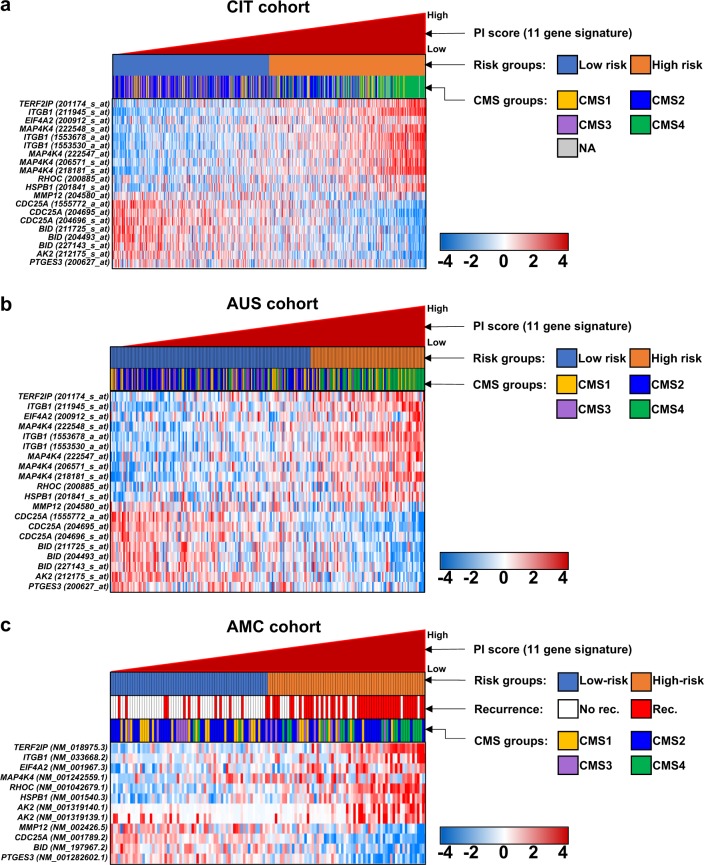
Comparison of subgroups stratified by the prognostic index (PI) based on an eleven gene signature and four subtypes derived from consensus molecular subtypes (CMS). Gene expression patterns are displayed by the PI in the (**a**) CIT, (**b**) AUS, and (**c**) AMC cohorts. Red and green colors reflect high and low expression levels, respectively

We carried out a comparative analysis of the PI system and CMS, for which a total of four subclassifications of CRC have been described by an international consortium^19^: CMS1 with hypermutation, microsatellite unstable and strong immune activation; CMS2 with WNT and MYC signaling activation; CMS3 with evident metabolic dysregulation; and CMS4 with TGF–β activation, stromal invasion and angiogenesis. When the CMS were compared with the PI system in the CIT cohort, most of the patients (116 of 126, 92.1%) in CMS4, in which CRC patients had distinct features of worse relapse-free and overall survival and tended to be diagnosed at more advanced stages (III and IV)^19^, were classified as high risk by the PI (Supplementary Fig. S4a). Among the CMS, 40.4% (36 of 89) of the patients in CMS1, associated with the activation of immune evasion and a higher histopathological grade, were also classified as a high-risk subgroup by the PI. Despite the predominant involvement of high-risk CRC patients in CMS4 and CMS1, CMS2, and CMS3 contained more low-risk CRCs than other subtypes: 35.6% (82 of 230) and 24.2% (16 of 66) of the high-risk CRCs were included in CMS2 and CMS3, respectively (Supplementary Fig. S4a). When patients were sorted by PI scores, higher-scoring CRC patients tended to be classified in CMS4 (Fig. 4a), indicating that the high-risk subgroup stratified by the PI could well reflect a molecular subtype of CRC showing a poor prognosis derived from the epithelial-to-mesenchymal transition (EMT)^19^.

We also applied the CMS classifier provided by the CMS consortium to the gene expression data from the AUS cohort. Similar to the CIT cohort, the vast majority of CMS4 patients were high-risk CRC patients (45 of 60, 75%; Supplementary Fig. S4b). We also observed that many high-risk CRC patients were contained in the CMS1 subgroup (23 of 51, 45.1%), whereas CMS2 and CMS3 included fewer high-risk patients with CRC (17.3% (13 of 75) and 7.5% (3 of 40), respectively), consistent with the CMS classification in the CIT cohort (Supplementary Fig. S4b). When sorting the patients based on PI scores, many higher-scoring CRC patients were classified into CMS4 (Fig. 4b), validating the prognostic relevance of the PI classification as an indicator of the poor prognosis of CRC patients.

Using an eleven gene signature, which makes up the PI system, we also calculated a PI for each patient in the AMC cohort. When the patients in the AMC cohort were classified into low- or high-risk subgroups by the PI, the frequencies of disease recurrence between the two risk subtypes were significantly different (Fisher’s exact test, *P* < 0.001, odds ratio = 4.759, 95% CI = 1.78–8.64; Fig. 4c), congruent with a hierarchical clustering analysis using the 1160 systemic recurrence-associated genes (Fig. 1). Comparing the expression levels of 11 genes between subgroups, we observed that these genes were significantly differentiated (two-sample *t*-tests, each *P* < 0.05; Supplementary Fig. S5). We also applied a CMS classifier to the RNA-seq data from the AMC cohort. When examining the frequencies of the PI-classified high-risk CRC patients involved in the CMS subgroups, we obtained proportions of 31% (9 of 29), 44.1% (26 of 59), 50% (9 of 18), and 87.5% (21 of 24) high-risk CRCs included in the CMS1, CMS2, CMS3, and CMS4 subgroups, respectively, demonstrating a similar high-risk patient involvement in CMS4 compared with the CIT and AUS cohorts (Supplementary Fig. S4c). Many high-scoring CRC patients sorted by the PI were obviously associated with CMS4, although a number of patients in the other CMS subgroups were predicted as high-risk CRCs in systemic recurrence (Fig. 4c).

Finally, we compared the genes between the PI and the CMS classifiers. When comparing 11 genes in the PI system and 273 genes in the CMS classifier based on a random forest prediction model^19^, interestingly, no common genes were found between two gene lists, despite similar prognostic behaviors between CRC patients with high PI scores and the CMS4 subgroup.

## Discussion

Extensive advancements in massive high-throughput technology have provided many insights into CRC. Beyond various investigations of molecular subtypes in CRC^13,23–25^, a consensus by an international consortium with great efforts to aggregate previously reported subtypes has also been reported^19^, which aimed to uncover the tumor heterogeneity and guide an appropriate treatment option for CRC. Despite these rigorous efforts, few reliable biomarkers have been identified for the precise prediction of disease recurrence in CRC, which is one of the unmet needs for the handling or treatment of CRC patients. Here, we collected a total of 130 primary CRCs with or without systemic recurrence and generated a transcriptome dataset based on RNA-seq analysis. Through gene expression profiling with pathway enrichment analysis, we identified 127 systemic recurrence-associated genes. Among these genes, eleven genes, *AK2*, *BID*, *CDC25A*, *EIF4A2*, *HSPB1*, *ITGB1*, *MAP4K4*, *MMP12*, *PTGES3*, *RHOC*, and *TERF2IP*, were identified as the best signature associated with recurrence-free survival of CRC in multiple patient cohorts, as validated by RT-PCR analysis. The PI, combined with these eleven genes, was strongly predictive of high-risk CRC and independent of currently available clinical indicators. The survival outcomes underscore the prognostic potential in stage III CRC patients and the specificity for adjuvant chemotherapy in stage II CRC patients.

The prediction of recurrence or metastasis is imperatively needed, as half of CRC cases are associated with fatal events. In this regard, we believe that molecularly driven methods will lead to the identification of recurrence-prone CRC patients and personalized treatment options, improving survival through a reduction in systemic recurrence. Recently, the CRC Subtyping Consortium (https://github.com/Sage-Bionetworks/crcsc) arranged six independent classifiers into four CMS. Similarly, our results in high-risk subgroups using the current PI criteria showed that CMS4 (mesenchymal type) incurred a worse overall and relapse-free survival and CMS1 (MSI-immune type) incurred a worse survival after relapse compared to those of the other subtypes^19^. Although several follow-up studies presented corresponding results, another study cautioned that the respective subtype, which did not consider tumor heterogeneity, could lead to individual patient classification into inappropriate subgroups^26^. However, single or subsets of marker(s) have been investigated to elucidate relapse predictors in CRC. MACC1 overexpression generally results in poor survival outcomes and is recognized as an independent prognostic indicator of relapse in CRC patients^19,27^.

Despite extensive investigations in CRC, it is still difficult to identify patients who will benefit the most or the least from adjuvant chemotherapy due to the heterogeneity of CRC. The benefits of adjuvant chemotherapy have been well established for patients with AJCC stage III cancer^21,22^, whereas its effectiveness in AJCC stage II patients remains controversial^5^. In several subset analyses in this study, the PI system demonstrated strong prognostic value in stage II and III CRC patients as well as significant predictive value for adjuvant chemotherapy in stage II CRC patients. In this study, the PI scores could identify a low-risk patient subgroup with stage II CRC who would benefit from adjuvant chemotherapy. In an interaction analysis, chemotherapy was strongly interactive with stage II and was significantly associated with a poorer outcome for patients in the low-risk subgroup as predicted by PI, whereas its effect was not significant for patients in the high-risk group in patients with stage II disease. First-line chemotherapy was clearly less effective in stage II tumors with PI underexpression than in all the other subgroups; therefore, other regimens, including targeted biologics, are recommended in the former group of patients.

The currently suggested PI system was generated from a combination of the expression levels of eleven genes, which were widely associated with the development and progression of CRC. The protein encoded by *AK2* is an enzyme found in the intermembrane space of the mitochondrion^28^. AK2 deficiency in humans causes hematopoietic defects and immunodeficiency^29,30^. *BID*, a BH3 interacting domain death agonist, encodes a protein that is a member of the BCL-2 family of cell death regulators, which are mediators of the mitochondrial damage induced by caspase-8. *BID* also has a number of major activities in the cell, such as apoptosis, cell death, activation, or proliferation. Although few associations between *BID* and cancer have been investigated, evaluations of *BID* in human colorectal diseases, such as colorectal adenoma or colitis, have been demonstrated^31,32^, suggesting that *BID* may be a possible mediator of CRC. *CDC25A*, cell division cycle 25A, is particularly degraded in response to DNA damage, resulting in cell cycle arrest and the transformation of fibroblasts interacting with oncogenic RAS^33^. *EIF4A2* encodes a protein, eukaryotic translation initiation factor 4A II, which is a component of the eIF4F complex during initiation of protein synthesis in the EIF2 signaling pathway. Although few cellular functions of *EIF4A2* have been discovered, positive associations between *EIF4A2* and various cancers, including CRC, have been investigated^34,35^, suggesting that *EIF4A2* may be a novel mediator of CRC recurrence. The protein encoded by *ITGB1* (Integrin beta-1) is a membrane receptor involved in cell adhesion and recognition in a variety of processes, including embryogenesis, hemostasis, tissue repair, the immune response and metastatic diffusion of tumor cells. Numerous investigations describing significantly positive correlations between *ITGB1* and various cancers, such as gastric^36^, breast^37^, and lung^38^ cancer, clearly support an aggressive characteristic of *ITGB1* in cancer. The previously reported functional roles of *ITGB1* in CRC cell lines, such as cell adhesion^39,40^, proliferation^41^, apoptosis^42^, and phosphorylation^43^, clearly support a contribution of *ITGB1* to CRC aggressiveness. *MAP4K4* encodes the protein MEK kinase kinase 4, which is associated with a wide range of physiological processes, including cell migration, proliferation and adhesion. The poor prognosis and disease progression of CRC are closely correlated with *MAP4K4* expression levels^44^. *RHOC* encodes a member of the Rho family of small GTPases (i.e., ras homolog family member C). *RHOC* is also involved in the mTOR signaling pathway, in which mTORC2 regulates cytoskeletal organization by the GTP loading of proteins of the Rho family^45^. *RHOC* overexpression is associated with tumor cell proliferation and metastasis and is especially involved in the CRC metastasis signaling pathway, underscoring the prognostic value of PI-containing *RHOC* expression. Among the 11 genes in the signature, *MMP12* (matrix metalloproteinase 12), *PTGES3* (prostaglandin E synthase 3), and *TERF2IP* (telomeric repeat-binding factor 2-interacting protein 1) are involved in some clinical activities^46^, but their clinical association with CRC has been poorly reported, suggesting that these genes might be novel markers in CRC prognosis.

Although the clinical relevance of our PI system was investigated across multiple patient CRC cohorts, there are several limitations to our study. Because of the short follow-up duration of the data in the AMC discovery cohort, the prognostic relevance of the 11 genes in the signature could not be confirmed in that cohort. The number of patients who received adjuvant chemotherapy was not sufficient to clearly determine the chemoresponse of the PI system, even though these results illustrated the chemospecificity in stage II CRC patients. In addition, as too few samples in an independent patient group were assessed in the PCR validation, it was difficult to determine the significance of the genes. The PI system should be rigorously validated in larger independent clinical cohorts with a longer follow-up time of the CRC patients.

In conclusion, we identified distinct prognostic subgroups defined by the expression of eleven genes in CRC. As a prognostic and a predictive indicator, the newly identified PI system may not only identify high-risk stage II and stage III CRC patients but also predict low-risk stage II CRC patients who benefit the least from adjuvant chemotherapy. For practical clinical use of the PI, however, more elaborate and rigorous validation steps are needed, which not only evaluate additional larger patient cohorts but also estimate detectability through a liquid biopsy.

## Supplementary information

Supplementary Information
