# Supplementary material for: A prognostic index based on an eleven gene signature to predict systemic recurrences in colorectal cancer
Source: Exp Mol Med. 2019 Oct 2;51(10):115. doi: 10.1038/s12276-019-0319-y (PMC6802642; doi:10.1038/s12276-019-0319-y)
Supplement: Supplementary file 1 — Supplementary Information [file 12276_2019_319_MOESM1_ESM.pdf]

Supplementary Information

Supplementary Table S1. Baseline characteristics of colorectal cancer patient cohorts.

Supplementary Table S2. Sequencing coverage of RNAseq data of colorectal cancer patient in the AMC cohort.

Supplementary Table S3. Primer sequence of the four genes for real-time reverse transcriptase polymerase chain reaction (RT-PCR) analysis.

Supplementary Table S4. 127 genes associated with systemic recurrence in colorectal cancer and involved in known canonical pathways.

Supplementary Table S5. Prognostic value estimation of 11 genes (17 unique probes) in colorectal cancer of the CIT cohort.

Supplementary Table S6. Univariate and multivariate Cox regression analysis of disease-free survival in colorectal cancer (CIT cohort).

Supplementary Figure S1. Pathway enrichments analysis of 1,160 genes associated with systemic recurrence in colorectal cancer.

Supplementary Figure S2. Comparison of expression levels between colorectal cancer (CRC) patients without recurrence and with recurrence.

Supplementary Figure S3. Interaction of the prognostic index (PI) classifier with adjuvant chemotherapy in patients with colorectal cancer in the combined cohort.

Supplementary Figure S4. Comparison of subgroup stratified by prognostic index (PI) based on a four gene signature and 4 subtypes derived by consensus molecular subtypes (CMS).

Supplementary Figure S5. Comparison of mRNA expression levels between the high-risk and low-risk subgroups of colorectal cancer (CRC) patients in the AMC cohort.

Supplementary Table S1. Baseline characteristics of colorectal cancer patient cohorts.

Variables	AMC cohort	CIT cohort	AUS cohort
Patients (n)	130	566	229
Gender, n (%)			
Male	79 (60.8)	310 (54.8)	123 (53.7)
Female	51 (39.2)	256 (45.2)	106 (46.3)
Age (years)			
Median	58	68	67
Range	25-80	22-97	26-92
Location, n (%)			
Colon	96 (73.8)	566 (100)	199 (86.9)
Rectum	34 (26.2)	0 (0)	30 (13.1)
NA			
AJCC Stage, n (%)			
I	7 (5.4)	37 (6.5)	44 (19.2)
II	46 (35.4)	264 (46.7)	94 (41.1)
III	28 (21.5)	205 (36.2)	91 (39.7)
IV	49 (37.7)	60 (10.6)	0
NA			
Adjuvant chemotherapy, n (%)			
Yes	74 (56.9)	233 (41.2)	87 (38.0)
No	56 (43.1)	316 (55.8)	142 (62.0)
NA		17 (3.0)	
Number of events	NA	177	50
Median DFS (months)	41.5	48.0*	38.5

*Median DFS of the CIT cohort was estimated excluding the patients with stage IV disease.

Abbreviations: AMC, Asan Medical Center; CIT, Cartes d'Identité des Tumeurs; AUS, Austrailian-US; NA, not available; AJCC, American Joint Committee on Cancer; DFS, disease-free survival.

Supplementary Table S2. Sequencing coverage of RNAseq data of colorectal cancer patient in the AMC cohort.

Patient ID	# of reads (mapped to human transcriptome)	Coverage (read depth)*
AMC-10	43,649,286	53.8X
AMC-11	74,154,710	91.4X
AMC-12	35,936,084	44.3X
AMC-13	34,082,474	42X
AMC-16	25,773,616	31.8X
AMC-17	25,732,538	31.7X
AMC-18	33,541,594	41.4X
AMC-19	48,069,992	59.3X
AMC-20	40,243,834	49.6X
AMC-21	31,763,854	39.2X
AMC-22	26,293,816	32.4X
AMC-23	35,445,998	43.7X
AMC-24	34,024,790	42X
AMC-2	49,359,434	60.9X
AMC-3	42,037,462	51.8X
AMC-5	29,612,092	36.5X
AMC-6	42,734,540	52.7X
AMC-7	43,261,008	53.3X
AMC-8	33,741,998	41.6X
AMC-9	33,575,516	41.4X
AMC-R100	44,753,090	55.2X
AMC-R101	31,565,796	38.9X
AMC-R102	56,572,456	69.8X
AMC-R103	26,587,314	32.8X
AMC-R104	39,748,456	49X
AMC-R109	37,661,984	46.4X
AMC-R110	36,112,974	44.5X
AMC-R111	36,857,724	45.4X
AMC-R112	23,960,682	29.5X
AMC-R113	34,606,430	42.7X
AMC-R114	29,000,834	35.8X
AMC-R116	29,229,630	36X
AMC-R117	34,734,432	42.8X
AMC-R120	34,866,090	43X
AMC-R121	33,130,120	40.8X
AMC-R122	39,061,010	48.2X

AMC-R123	34,586,654	42.6X
AMC-R124	38,121,726	47X
AMC-R125	59,626,460	73.5X
AMC-R126	84,611,802	104.3X
AMC-R127	63,025,038	77.7X
AMC-R128	73,077,702	90.1X
AMC-R129	67,299,806	83X
AMC-R12	20,909,966	25.8X
AMC-R131	79,165,012	97.6X
AMC-R132	56,227,794	69.3X
AMC-R133	56,226,796	69.3X
AMC-R134	44,786,924	55.2X
AMC-R135	46,849,508	57.8X
AMC-R136	48,978,760	60.4X
AMC-R137	44,570,228	55X
AMC-R138	59,928,646	73.9X
AMC-R139	35,617,924	43.9X
AMC-R140	43,338,380	53.4X
AMC-R141	21,116,858	26X
AMC-R142	59,409,850	73.2X
AMC-R143	55,825,718	68.8X
AMC-R146	47,314,858	58.3X
AMC-R147	60,841,942	75X
AMC-R148	45,125,934	55.6X
AMC-R150	66,052,326	81.4X
AMC-R155	44,232,252	54.5X
AMC-R156	27,280,606	33.6X
AMC-R157	24,331,206	30X
AMC-R158	39,085,378	48.2X
AMC-R159	48,535,030	59.8X
AMC-R160	46,848,228	57.8X
AMC-R161	62,437,828	77X
AMC-R162	46,820,694	57.7X
AMC-R167	52,022,226	64.1X
AMC-R168	55,904,648	68.9X
AMC-R170	51,938,792	64X
AMC-R183	39,631,792	48.9X
AMC-R1	27,374,584	33.8X
AMC-R212	40,781,512	50.3X

AMC-R223	32,874,744	40.5X
AMC-R225	38,208,514	47.1X
AMC-R229	33,345,508	41.1X
AMC-R230	39,738,954	49X
AMC-R231	35,327,706	43.6X
AMC-R232	33,206,734	40.9X
AMC-R233	27,263,572	33.6X
AMC-R235	43,647,832	53.8X
AMC-R23	39,539,554	48.8X
AMC-R241	31,371,896	38.7X
AMC-R243	49,375,248	60.9X
AMC-R245	26,810,862	33.1X
AMC-R246	57,862,846	71.3X
AMC-R248	31,147,338	38.4X
AMC-R249	27,359,856	33.7X
AMC-R253	23,047,098	28.4X
AMC-R256	31,305,612	38.6X
AMC-R258	58,123,106	71.7X
AMC-R259	24,588,008	30.3X
AMC-R25	24,007,290	29.6X
AMC-R261	22,309,814	27.5X
AMC-R262	50,536,312	62.3X
AMC-R265	27,433,352	33.8X
AMC-R267	35,543,332	43.8X
AMC-R270	21,860,468	27X
AMC-R278	192,689,598	237.6X
AMC-R284	60,847,084	75X
AMC-R289	59,648,498	73.5X
AMC-R290	39,904,956	49.2X
AMC-R293	36,261,234	44.7X
AMC-R295	31,300,168	38.6X
AMC-R2	32,299,922	39.8X
AMC-R34	21,503,562	26.5X
AMC-R39	28,190,836	34.8X
AMC-R49	22,327,938	27.5X
AMC-R4	25,472,560	31.4X
AMC-R50	44,599,508	55X
AMC-R52	28,005,226	34.5X
AMC-R54	29,578,954	36.5X

AMC-R57	25,556,810	31.5X
AMC-R75	28,887,652	35.6X
AMC-R85	33,713,296	41.6X
AMC-R86	31,833,990	39.2X
AMC-R87	33,937,842	41.8X
AMC-R88	34,382,346	42.4X
AMC-R8	23,406,990	28.9X
AMC-R90	32,168,716	39.7X
AMC-R91	40,218,260	49.6X
AMC-R92	33,823,164	41.7X
AMC-R93	31,815,602	39.2X
AMC-R94	29,728,262	36.7X
AMC-R95	38,822,344	47.9X
AMC-R97	31,317,186	38.6X
AMC-R98	31,693,584	39.1X
AMC-R99	29,540,420	36.4X

*Read depths were estimated on the human transcriptome, whose length is approximately 82 Megabases.

Supplementary Table S3. Primer sequence of the four genes for real-time reverse transcriptase polymerase chain reaction (RT-PCR) analysis.

Gene	Strand	Sequence
ITGB1	F	5'- CGAGGTCATGGTTCATGTTG-3'
	R	5'-ATCGGATTTTCTTGCGTGTC-3'
RHOC	F	5'-ACAGCAGGGCAGGAAGACTA-3'
	R	5'-TTCATCTTGGCCAGCTCTCT-3'
EIF4A2	F	5'-TGACCCTTGAAGGAATCAAAC-3'
	R	5'-GTCCATGTCACCATGCAGAG-3'
BID	F	5'-ACAGCATGGACCGTAGCATC-3'
	R	5'-AAAGACATCACGGAGCAAGG-3'

Abbreviations: F, forward; R, reverse.

Supplementary Table S4. 127 genes associated with systemic recurrence in colorectal cancer and involved in known canonical pathways.

Symbol	*log2 transformed fold change in expression	$^{\Psi}$*r*-value	$^{\Psi}$*p*-value
RHOC	1.761	0.37	<0.001
RPL8	1.479	0.358	<0.001
FGFRL1	1.429	0.464	<0.001
JUND	1.263	0.381	<0.001
ALB	1.1	0.509	<0.001
HSPB1	0.967	0.33	<0.001
HP	0.95	0.442	<0.001
HDAC7	0.923	0.327	<0.001
AKT2	0.855	0.303	<0.001
CEBPB	0.834	0.353	<0.001
APOA2	0.812	0.3	<0.001
STK11	0.806	0.383	<0.001
JUN	0.771	0.34	<0.001
NOTCH1	0.755	0.399	<0.001
STX4	0.705	0.341	<0.001
SPHK1	0.675	0.3	<0.001
RPS17	0.658	0.326	<0.001
MKNK2	0.632	0.392	<0.001
MAGI1	0.627	0.329	<0.001
IKBKG	0.617	0.323	<0.001
RPL15	0.598	0.39	<0.001
PIK3R2	0.586	0.361	<0.001
FGA	0.564	0.384	<0.001
SREBF1	0.549	0.356	<0.001
HRAS	0.543	0.326	<0.001
ATF2	0.528	0.372	<0.001
ORM1	0.526	0.323	<0.001
ELAVL1	0.504	0.421	<0.001
FGG	0.495	0.394	<0.001
EEF2	0.476	0.301	<0.001
GADD45G	0.467	0.308	<0.001
SRGN	0.433	0.354	<0.001
YWHAZ	0.402	0.307	<0.001
BORCS8-MEF2B	0.4	0.314	<0.001
MLXIPL	0.396	0.301	<0.001
APOC3	0.392	0.384	<0.001
SIRPA	0.378	0.343	<0.001
APOA1	0.374	0.456	<0.001
BRAF	0.348	0.39	<0.001
MAP2K2	0.344	0.354	<0.001

CASP7	0.339	0.362	<0.001
HPX	0.336	0.32	<0.001
EIF4A2	0.334	0.328	<0.001
TERF2IP	0.329	0.422	<0.001
RXRB	0.307	0.323	<0.001
CYP2E1	0.304	0.339	<0.001
VTN	0.299	0.345	<0.001
CAPN10	0.297	0.341	<0.001
PTPN11	0.281	0.308	<0.001
AK2	0.275	0.441	<0.001
PLAGL1	0.248	0.337	<0.001
FOXO6	0.245	0.33	<0.001
PRKAR2A	0.24	0.325	<0.001
MEF2A	0.234	0.339	<0.001
APC	0.231	0.367	<0.001
RPL14	0.21	0.463	<0.001
WNT6	0.21	0.308	<0.001
CCNK	0.208	0.313	<0.001
SPRY2	0.181	0.325	<0.001
EIF3F	0.176	0.389	<0.001
ITGB1	0.175	0.313	<0.001
IL6R	0.172	0.346	<0.001
MAP4K4	0.168	0.306	<0.001
HRG	0.163	0.36	<0.001
RPL4	0.134	0.316	<0.001
CACNA1C	0.072	0.306	<0.001
RPL34	0.071	0.418	<0.001
FHIT	0.065	0.312	<0.001
FCGR3A	0.063	-0.371	<0.001
RAPGEF3	0.056	0.315	<0.001
CACNA1A	0.038	0.307	<0.001
FCGR1A	0.03	-0.342	<0.001
RPL31	0.03	0.34	<0.001
FCER1G	-0.033	-0.303	<0.001
TP53	-0.046	-0.302	<0.001
PRF1	-0.129	-0.327	<0.001
IL2RB	-0.133	-0.304	<0.001
MAPK8	-0.229	-0.365	<0.001
CSF2RA	-0.259	-0.351	<0.001
FAU	-0.273	-0.317	<0.001
IFNG	-0.292	-0.378	<0.001
MMP12	-0.311	-0.336	<0.001
ATG13	-0.312	-0.364	<0.001

IRF7	-0.32	-0.366	<0.001
STAT2	-0.331	-0.33	<0.001
FASLG	-0.339	-0.441	<0.001
MYL6	-0.34	-0.327	<0.001
CD40	-0.353	-0.399	<0.001
PRKAG1	-0.356	-0.313	<0.001
HLA-DRA	-0.368	-0.312	<0.001
BID	-0.371	-0.336	<0.001
IFI35	-0.378	-0.347	<0.001
BAK1	-0.398	-0.33	<0.001
CHUK	-0.398	-0.33	<0.001
HLA-DMB	-0.42	-0.326	<0.001
CAPNS1	-0.436	-0.304	<0.001
ZBP1	-0.438	-0.325	<0.001
TERF1	-0.472	-0.319	<0.001
RRM2B	-0.516	-0.337	<0.001
SH2D2A	-0.52	-0.35	<0.001
PPP2R2A	-0.54	-0.3	<0.001
MGST3	-0.548	-0.369	<0.001
CPT2	-0.56	-0.323	<0.001
RPL17	-0.565	-0.308	<0.001
PTGES3	-0.613	-0.35	<0.001
BBC3	-0.67	-0.403	<0.001
CD3D	-0.678	-0.341	<0.001
EIF4E	-0.717	-0.382	<0.001
IRF3	-0.724	-0.342	<0.001
BCL2L1	-0.757	-0.316	<0.001
MLST8	-0.818	-0.37	<0.001
IL18	-0.828	-0.316	<0.001
CDC25A	-0.88	-0.347	<0.001
AGO1	-0.936	-0.362	<0.001
E2F5	-0.96	-0.335	<0.001
EIF2B3	-0.983	-0.452	<0.001
BAX	-1.135	-0.335	<0.001
INPPL1	-1.159	-0.305	<0.001
SRF	-1.188	-0.334	<0.001
RPS9	-1.214	0.309	<0.001
IRAK1	-1.22	-0.373	<0.001
BCAR1	-1.25	-0.317	<0.001
SLC35A2	-1.273	-0.361	<0.001
EIF2AK3	-1.593	-0.395	<0.001
JAK1	-1.758	-0.381	<0.001
MMP2	-2.17	-0.321	<0.001

CAB39	-2.857	-0.336	<0.001

*Positive fold change value indicates higher expression of gene in the cluster 1 than in the cluster 2 in Fig. 1.
$^{\Psi}r$ and *p*-values were obtained by point-biserial correlation test between systemic recurrence and gene expression levels.

Supplementary Table S5. Prognostic value estimation of 11 genes (17 unique probes) in colorectal cancer of the CIT cohort.

Probe_ID	Symbol	Coefficient	*z*-score	*p*-value	HR (95% CI)
212175_s_at	*AK2*	-0.536	-3.715	<0.001	0.585 (0.441-0.776)
204493_at	*BID*	-0.396	-2.759	0.006	0.673 (0.508-0.892)
227143_s_at	*BID*	-0.504	-2.835	0.005	0.604 (0.426-0.856)
1555772_a_at	*CDC25A*	-0.233	-2.733	0.006	0.792 (0.67-0.936)
204695_at	*CDC25A*	-0.363	-3.343	0.001	0.695 (0.562-0.86)
200912_s_at	*EIF4A2*	0.543	2.616	0.009	1.722 (1.146-2.587)
201841_s_at	*HSPB1*	0.319	3.219	0.001	1.375 (1.133-1.67)
1553530_a_at	*ITGB1*	0.408	3.439	0.001	1.504 (1.192-1.898)
1553678_a_at	*ITGB1*	0.470	3.431	0.001	1.6 (1.223-2.093)
211945_s_at	*ITGB1*	0.758	3.696	<0.001	2.135 (1.428-3.191)
218181_s_at	*MAP4K4*	0.352	3.056	0.002	1.422 (1.135-1.782)
222547_at	*MAP4K4*	0.363	3.119	0.002	1.438 (1.145-1.807)
222548_s_at	*MAP4K4*	0.539	2.97	0.003	1.714 (1.201-2.446)
204580_at	*MMP12*	-0.133	-2.693	0.007	0.875 (0.794-0.964)
200627_at	*PTGES3*	-0.696	-2.632	0.008	0.499 (0.297-0.837)
200885_at	*RHOC*	0.330	2.144	0.008	1.39 (1.029-1.879)
201174_s_at	*TERF2IP*	0.887	4.069	<0.001	2.428 (1.584-3.721)

Abbreviations: HR, hazard ratio; CI, confidence interval.

Supplementary Table S6. Univariate and multivariate Cox regression analysis of disease-free survival in colorectal cancer (CIT cohort).

Variable	Univariate			Multivariate			Multivariate (*step*[*])		
	n	HR (95% CI)	*P*-value	n	HR (95% CI)	*P*-value	n	HR (95% CI)	*P*-value
**Gender (male vs. female)**	556	0.786 (0.582 - 1.062)	0.116	455	0.776 (0.546 - 1.103)	0.157	455	0.765 (0.541 - 1.081)	0.129
**Age (<75 or ≥75)**	556	0.947 (0.681 - 1.316)	0.744		1.145 (0.76 - 1.723)	0.518			
**AJCC Stage (I, II, III, or IV)**	556	2.687 (2.172 - 3.325)	< 0.001		2.092 (1.586 - 2.758)	< 0.001		2.035 (1.582 - 2.619)	< 0.001
**Location (distal, proximal, or rectum)**	556	0.778 (0.57 - 1.063)	0.115		0.811 (0.558 - 1.178)	0.271			
**Chemotherapy (No or Yes)**	540	1.809 (1.326 - 2.468)	< 0.001		0.943 (0.628 - 1.414)	0.775			
**MMR (pMMR or dMMR)**	510	0.355 (0.187 - 0.672)	0.001		0.637 (0.312 - 1.3)	0.215		0.593 (0.299 - 1.178)	0.136
**CMS subtype (CMS1, 2, 3, or 4)**	510	1.363 (1.178 - 1.577)	< 0.001		1.168 (0.975 - 1.399)	0.093		1.153 (0.964 - 1.378)	0.118
**PI (low-risk or high-risk[**])**	556	1.817 (1.343 - 2.458)	< 0.001		1.702 (1.186 - 2.443)	0.004		1.709 (1.194 - 2.447)	0.003

[*]A backward-forward step procedure was applied to optimize the multivariate model with the most informative variables.
[**]Predicted outcome in Fig. 2b, c was used for analysis.
Abbreviations: HR, hazard ratio; CI, confidence interval; MMR, DNA mismatch repair; dMMR, deficient MMR; pMMR, proficient MMR; CMS, consensus molecular subtype; PI, prognostic index.

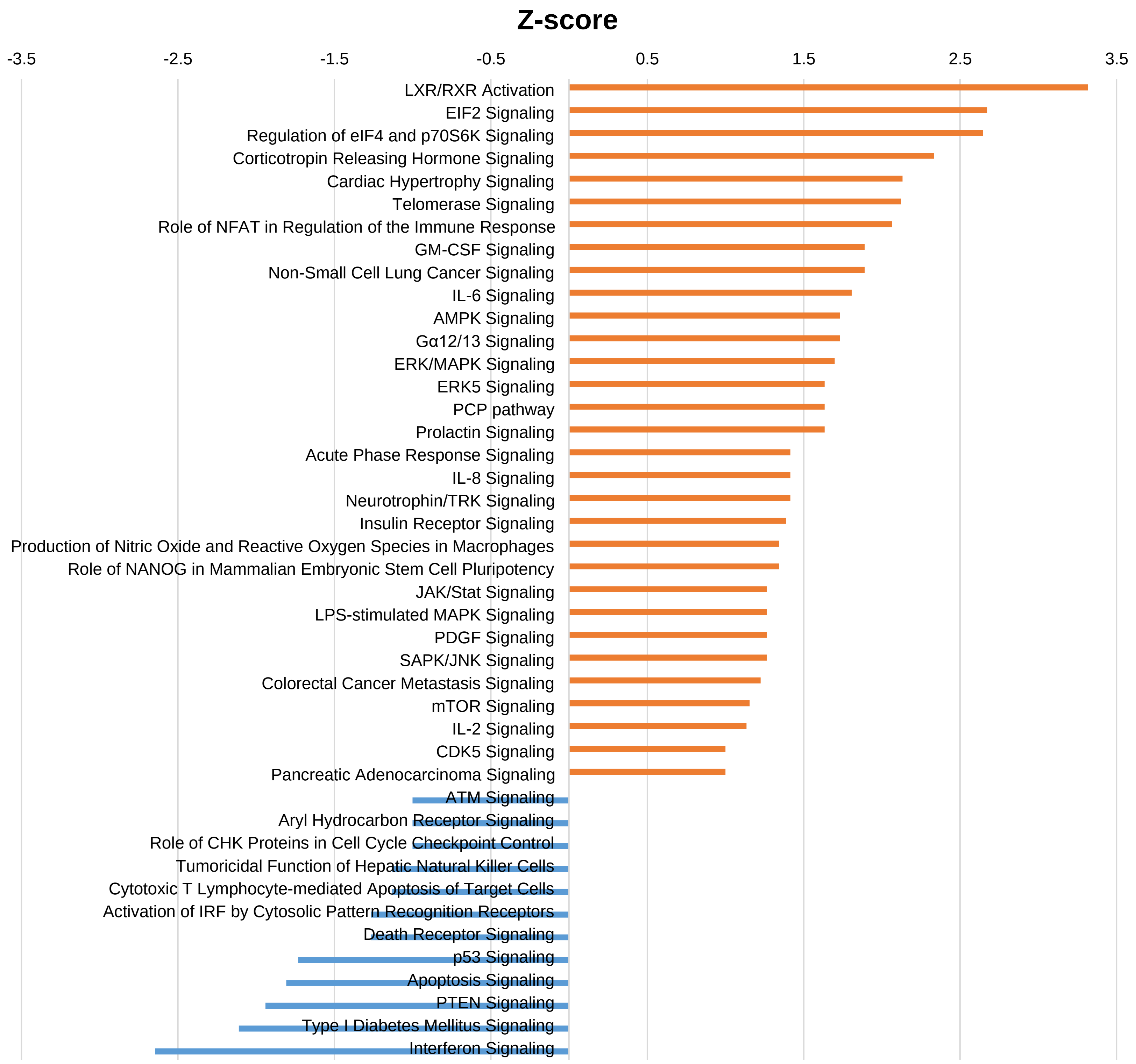

Supplementary Figure S1. Pathway enrichments analysis of 1,160 genes associated with systemic recurrence in colorectal cancer.

Classification enrichment was determined using Ingenuity Pathway Analysis software. The significantly activated or inhibited pathways were selected by $P < 0.05$ and Z-score > |1|. A positive or negative activation Z-score implies that a signaling pathway was activated or inhibited, respectively. P-values were obtained by Fisher-exact tests.

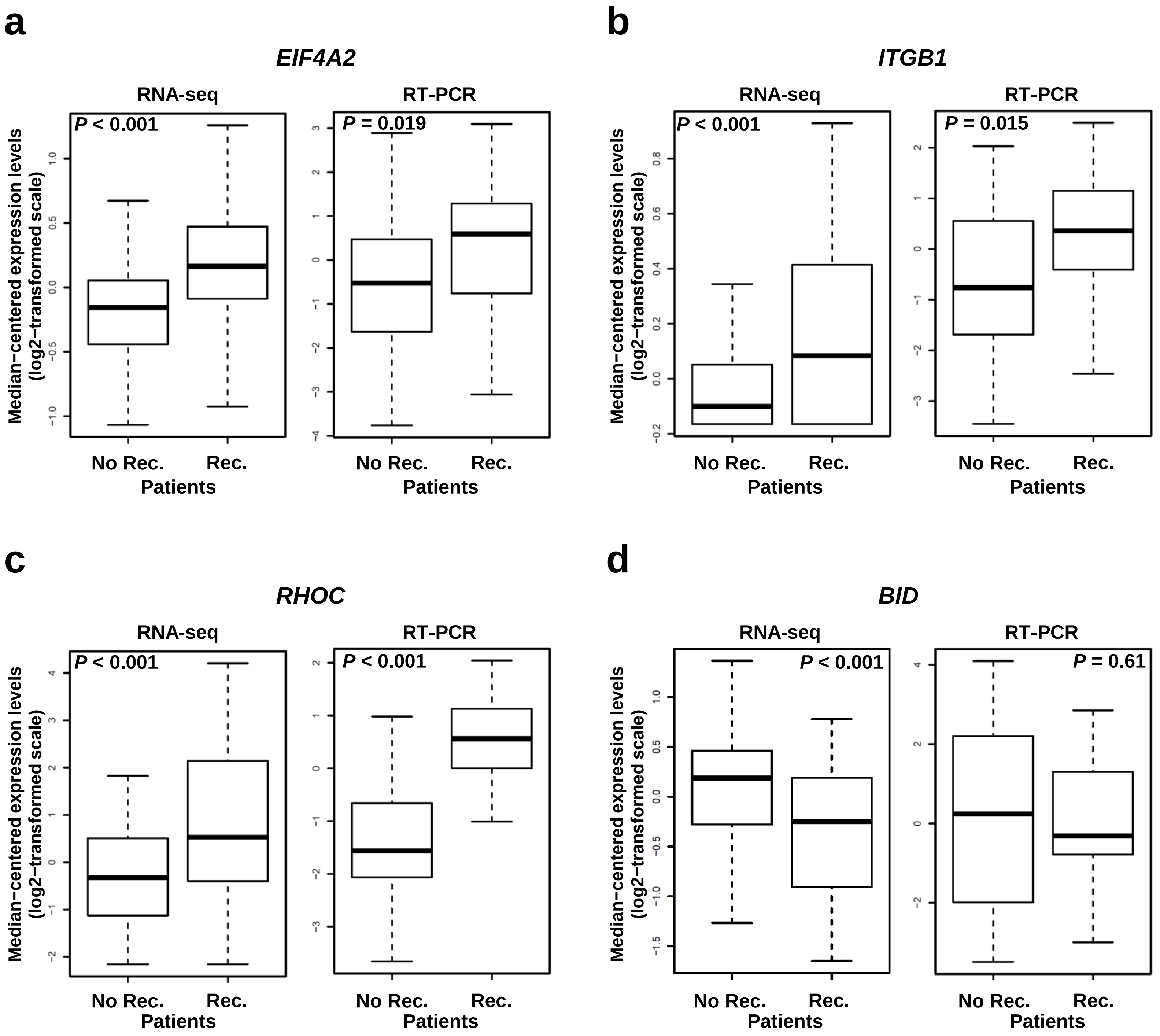

Supplementary Figure S2. Comparison of expression levels between colorectal cancer (CRC) patients without recurrence and with recurrence.

Left and right panels of each sub-figure displayed using expression data generated by RNA-seq and RT-PCR analyses, respectively. Two group box plot comparing expression levels of (a) *EIF4A2*, (b) *ITGB1*, (c) *RHOC*, and (d) *BID* in CRC patients without and with recurrence. *P*-value was obtained by two-sample t-test between CRC sample groups. No Rec, patients without recurrence. Rec, patients with recurrence.

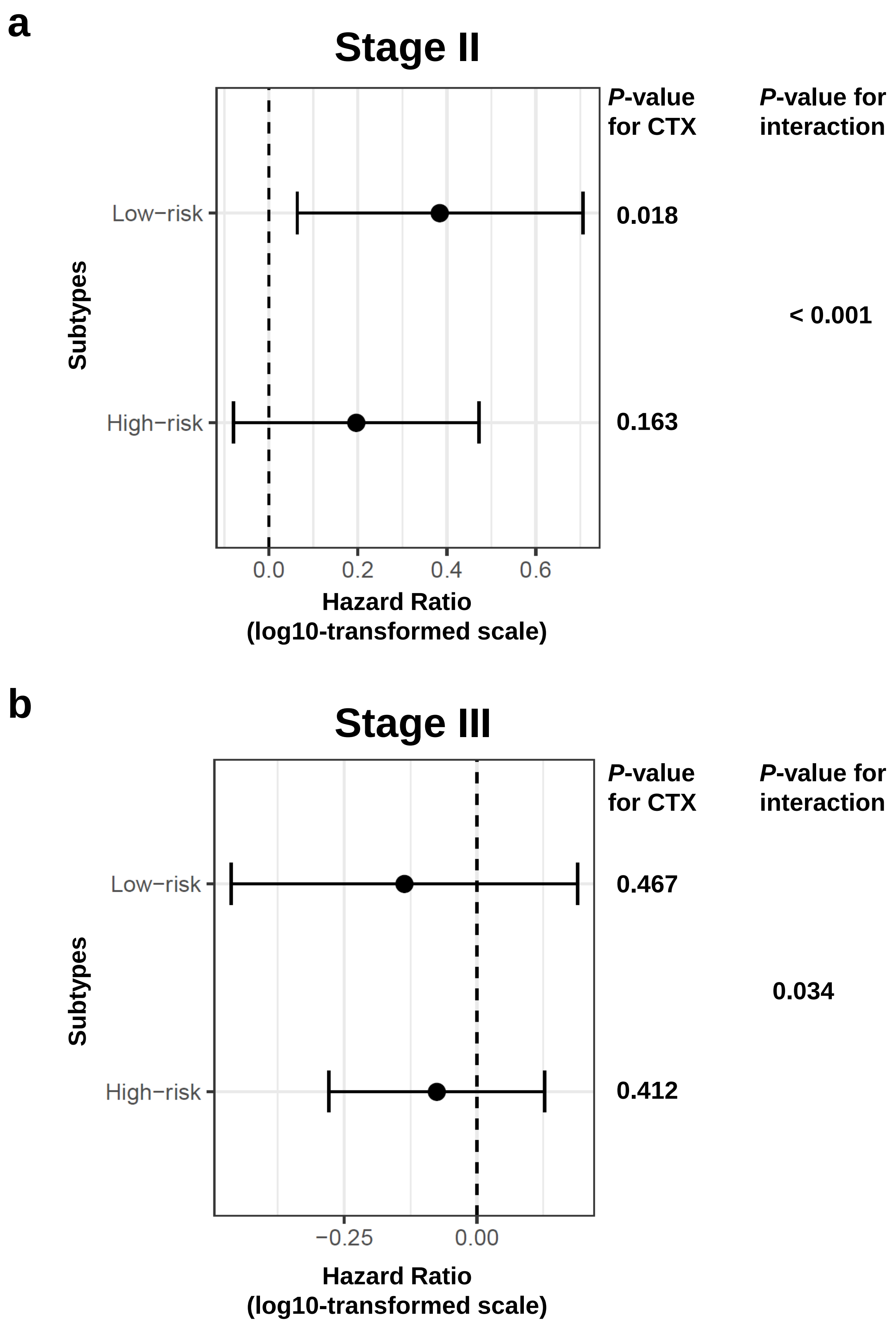

Supplementary Figure S3. Interaction of the prognostic index (PI) classifier with adjuvant chemotherapy in patients with colorectal cancer in the combined cohort.

Cox proportional hazard model was used to analyze interaction between the PI system and adjuvant chemotherapy. Solid line represent 95% confidence interval of hazard ratios. CTX, chemotherapy.

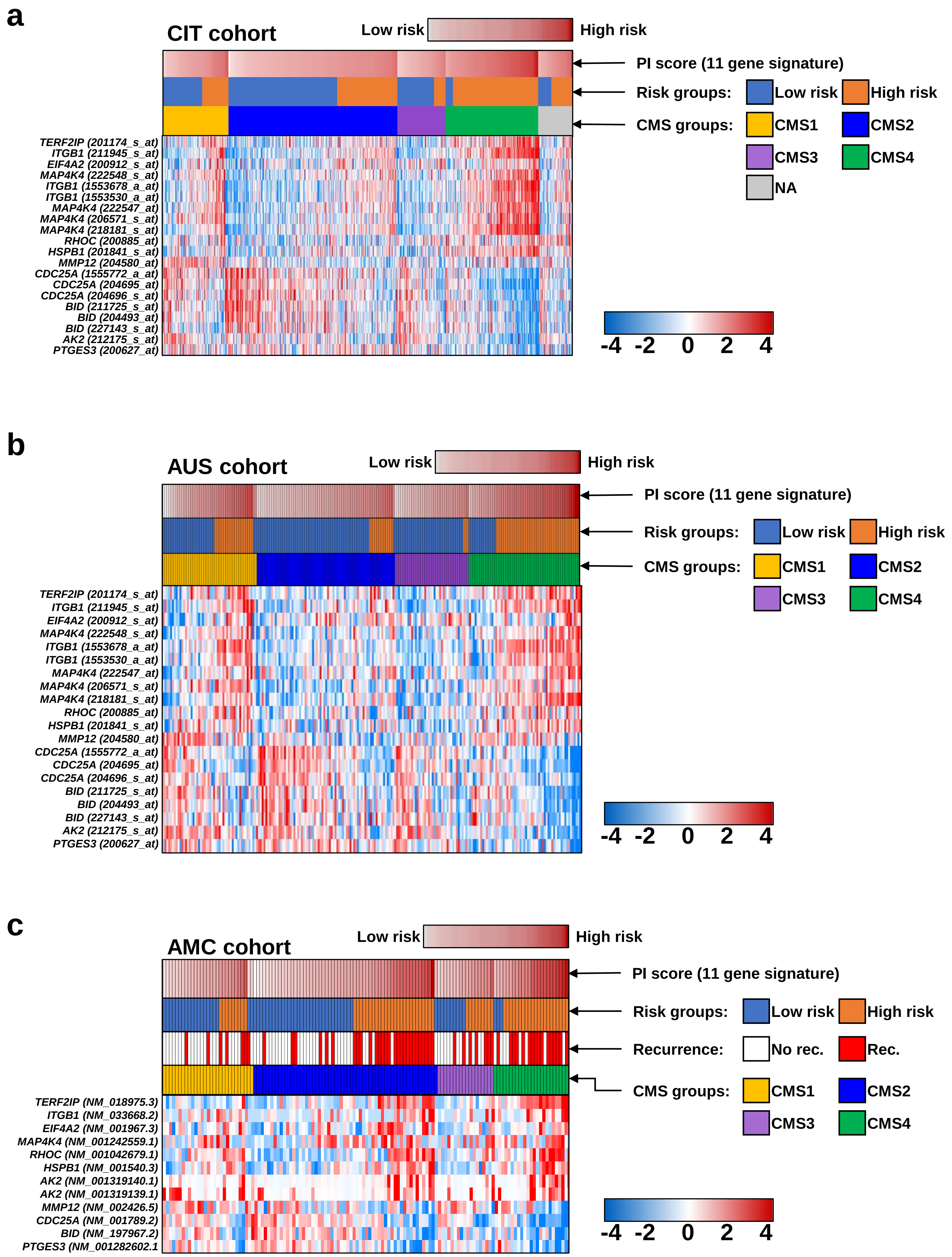
a
CIT cohort
Low risk
High risk
PI score (11 gene signature)
Risk groups:
Low risk
High risk
CMS groups:
CMS1
CMS2
CMS3
CMS4
NA
TERF2IP (201174_s_at)
ITGB1 (211945_s_at)
EIF4A2 (200912_s_at)
MAP4K4 (222548_s_at)
ITGB1 (1553678_a_at)
ITGB1 (1553530_a_at)
MAP4K4 (222547_at)
MAP4K4 (206571_s_at)
MAP4K4 (218181_s_at)
RHOC (200885_at)
HSPB1 (201841_s_at)
MMP12 (204580_at)
CDC25A (1555772_a_at)
CDC25A (204695_at)
CDC25A (204696_s_at)
BID (211725_s_at)
BID (204493_at)
BID (227143_s_at)
AK2 (212175_s_at)
PTGES3 (200627_at)
-4 -2 0 2 4
b
AUS cohort
Low risk
High risk
PI score (11 gene signature)
Risk groups:
Low risk
High risk
CMS groups:
CMS1
CMS2
CMS3
CMS4
TERF2IP (201174_s_at)
ITGB1 (211945_s_at)
EIF4A2 (200912_s_at)
MAP4K4 (222548_s_at)
ITGB1 (1553678_a_at)
ITGB1 (1553530_a_at)
MAP4K4 (222547_at)
MAP4K4 (206571_s_at)
MAP4K4 (218181_s_at)
RHOC (200885_at)
HSPB1 (201841_s_at)
MMP12 (204580_at)
CDC25A (1555772_a_at)
CDC25A (204695_at)
CDC25A (204696_s_at)
BID (211725_s_at)
BID (204493_at)
BID (227143_s_at)
AK2 (212175_s_at)
PTGES3 (200627_at)
-4 -2 0 2 4
c
AMC cohort
Low risk
High risk
PI score (11 gene signature)
Risk groups:
Low risk
High risk
Recurrence:
No rec.
Rec.
CMS groups:
CMS1
CMS2
CMS3
CMS4
TERF2IP (NM_018975.3)
ITGB1 (NM_033668.2)
EIF4A2 (NM_001967.3)
MAP4K4 (NM_001242559.1)
RHOC (NM_001042679.1)
HSPB1 (NM_001540.3)
AK2 (NM_001319140.1)
AK2 (NM_001319139.1)
MMP12 (NM_002426.5)
CDC25A (NM_001789.2)
BID (NM_197967.2)
PTGES3 (NM_001282602.1
-4 -2 0 2 4

Supplementary Figure S4. Comparison of subgroup stratified by prognostic index (PI) based on a four gene signature and 4 subtypes derived by consensus molecular subtypes (CMS).

Gene expression patterns were displayed order by the CMS subtypes in (a) CIT, (b) AUS, and (c) AMC cohorts. The red and green colors reflect high and low expression levels, respectively.

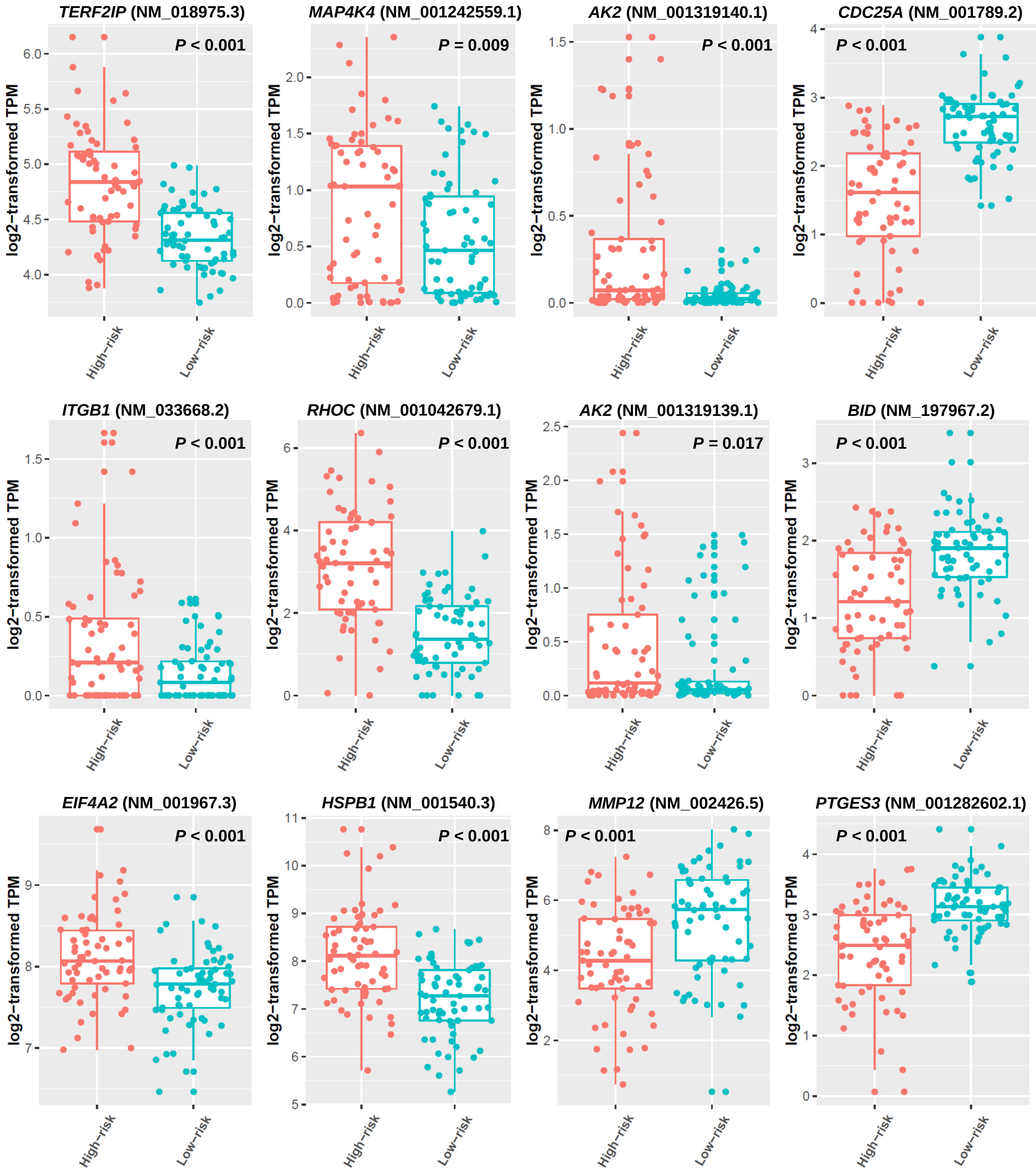

Supplementary Figure S5. Comparison of mRNA expression levels between the high-risk and low-risk subgroups of colorectal cancer (CRC) patients in the AMC cohort.

The prognostic index (PI) system divided CRC patients into high- or low-risk subgroups. Two group box plots comparing expression levels of 11 genes involved in the PI system were illustrated. *P*-values were obtained by two-sample t-tests. AMC, Asan Medical Center.
